# Untargeted Safety Pharmacology Screen of Blood-Activating and Stasis-Removing Patent Chinese Herbal Medicines Identified Nonherbal Ingredients as a Cause of Organ Damage in Experimental Models

**DOI:** 10.3389/fphar.2019.00993

**Published:** 2019-09-12

**Authors:** Xinyan Liu, Rui Shao, Xinyue Yang, Guangxu Xiao, Shuang He, Yuxin Feng, Yan Zhu

**Affiliations:** ^1^Tianjin State Key Laboratory of Modern Chinese Medicine, Tianjin University of Traditional Chinese Medicine, Tianjin, China; ^2^Research and Development Center of TCM, Tianjin International Joint Academy of Biotechnology & Medicine, Tianjin, China; ^3^Molecular Cardiology Research Institute, Tufts Medical Center and Tufts University School of Medicine, Boston, MA, United States

**Keywords:** safety pharmacology, Chinese herbal medicine, blood-activating and stasis-removing medicines, herbal–drug interaction, organ damage, toxicity

## Abstract

Blood activation and stasis removal from circulation is a central principle for treatment of syndromes related to cerebral and cardiovascular diseases in Chinese herbal medicine. However, blood-activating and stasis-removing patent Chinese herbal medicine (BASR-pCHM) widely used with or without prescription in China and elsewhere are highly variable in composition and manufacture standard, making their safety assessment a challenging task. We proposed that an integrated evaluation of multiple toxicity parameters of BASR-pCHM would provide critical reference and guidelines for their safe clinical application. Examination of standardized extracts from 58 compound BASR-pCHM *in vivo* in VEGFR2-luc mice and *in vitro* in cardiac, renal, and hepatic cells identified Naoluotong capsule (NLTC) as a potent organ/cell damage inducer. Composition analysis revealed that NLTC was the one that contained nonherbal ingredients among the BASR-pCHM collection. *In vivo* and *in vitro* experiments confirmed that NLTC, as well as its chemical supplement tolperisone hydrochloride, caused organ and cell damage by reducing cell viability, mitochondrial mass/activity, while the NLTC herbal components did not. Taken together, our study showed that safety evaluation of patent herbal medicines already on market is still necessary and urgently needed. In addition, chemical/herbal interactions should be considered as an important contributor of potential toxicity when evaluating the safety of herbal medicine.

## Introduction

Chinese herbal medicine (CHM) has been used in treating various diseases and maintaining health for Chinese people for more than 2,000 years (Hao et al., 2015). With both unique theories and rich experience, it is increasingly recognized worldwide (Pittler and Ernst, 2003; Fang and Xiong, 2019). Modernization of CHM boosted the manufacture and application of patent CHM (pCHM), which accounts up to one-third total drugs distributed and used on the market in China (Wang L. et al., 2015; Wang et al., 2017). Importantly, patients who used more types of pCHM tended to use much less Western medicine recommended by current guidelines. However, the safety concern of pCHM has constantly attracted attention from both health-care professionals and the public, as some pCHMs have been reported to have toxic side effects (Teschke and Eickhoff, 2015; Wang et al., 2018). Moreover, since CHM formula often consists of dozens of ingredients with innumerous chemical molecules, objective, and quantitative safety evaluation criteria for pCHM are difficult to establish (Teschke and Eickhoff, 2015).

Blood-activating and stasis-removing (BASR) CHMs are used mainly for treating cardiovascular diseases (Zhang, 2018). As an important class of CHM following the principle of synergy and toxin removal, many proprietary BASR pCHMs have been produced by standardized industrial procedures and are used for treating cardiovascular and cerebrovascular patients. The efficacy of the BASR pCHMs has been proven in both animal experiments and in the clinics. However, pCHMs belonging to the BASR category are vast, and the disease distinctions by individual members are vaguely defined. We have previously investigated pharmacological efficacy of BASR-pCHMs on the index of inhibiting platelet aggregation and dilatation of arterial tubes with the adult doses *in vivo*. Therefore, the collection and preparation process of our drugs are consistent with the previous pharmacodynamic experiments (Lu et al., 2015; Meng et al., 2016; Wang et al., 2017).

Adverse outcome pathways (AOPs) are a toxicological approach developed recently to connect mechanistic information to apical endpoints for regulatory purposes. It links a molecular initiating event to the adverse outcome *via* key events, in a way specified by key event relationships (Leist et al., 2017). The AOP approach gathers toxicity data using high-throughput cell- and biochemical-based tests to evaluate the combined data to predict potential toxic effects. Since the AOP concept is highly adaptable for CHM, we proposed that a systematic evaluation of multiple toxicity parameters of patent BASR-CHM would provide critical reference for their safe clinical application and briefly explore the material basis of typical adverse drug reactions.

## Materials and Methods

### Reagents and Drugs

Dulbecco’s modified Eagle’s medium (DMEM) and other cell culture supplies were purchased from Gibco (Grand Island, NY, USA). Hoechst 33342 was obtained from Invitrogen (Eugene, USA). Doxorubicin hydrochloride (Dox, batch number 20120205) was from Meilun Biotech Co. Ltd, (Dalian, China). Hematoxylin and eosin (H&E) staining kit (C0105) was purchased from Beyotime Biotechnology (Shanghai, China). ELISA kits for lactate dehydrogenase (LDH), aspartate aminotransferase (AST), creatine kinase-MB (CK-MB), and serum creatinine (Scr) were purchased from Biosino Bio-Technology and Science Inc. (Beijing, China). Tolperisone hydrochloride (batch number HY.B1139, purity >98% (Patel et al., 2011), was purchased from MedChem Express Corporation (New Jersey, USA). Methyl hesperidin (batch number 111580, purity >98%) was purchased from China Pharmaceutical and Biological Products Inspection Institute (Beijing, China). Vitamin B6 (batch number SV8110, purity >98%) was purchased from Beijing Solarbio Co., Ltd. (Beijing, China). KH_2_PO_4_, acetonitrile, methanol, and sodium heptane sulfonate (C_7_H_15_NaO_3_S) were purchased from Sigma (St Louis, Missouri, USA). Fifty-eight BASR-pCHMs, including Naoluotong capsule (NLTC), were purchased from certified local pharmacies and prepared as we described previously (Meng et al., 2016, detailed information in **Supplemental Data Sheet 1** and **2**).

### Animals and Drug Administration

VEGFR2-Luc mice, in which a luciferase reporter (luc) is under the control of the murine VEGFR2 promoter (Zhang et al., 2004), were obtained by breeding transgenic colonies maintained under specific pathogen-free (SPF) conditions at Tianjin International Joint Academy of Biotechnology and Medicine (TJAB). Institute of Cancer Research (ICR) mice were purchased from Beijing Hua Fu Kang (HFK) Bioscience Co. Ltd (License number SCXK-[jun] 2014-001schke3) and maintained under SPF conditions at TJAB. All animal experiments were performed in accord with the international regulations, following the guidelines of Tianjin University of TCM Animal Research Committee (TCM-LAEC2014004) and approved by the animal care and use committee of TJAB (No. TJU20160021).

All BASR-pCHMs were weighed, dissolved in ultrapure water, sonicated for 30 min, and filtered through a 0.22-µm polyvinylidene fluoride filter into stock solutions at a concentration of 10 mg/ml (Meng et al., 2016). Tolperisone hydrochloride, methyl hesperidin, and vitamin B6 were freshly prepared in ddH_2_O at the time of use.

### Animal Administration and Treatment

In order to evaluate whether the effects on the tissues at the clinical dose of commercial pCHMs that are the same as BASR function are consistent with each other, 58 BASR-pCHMs were administered VEGFR2-Luc mice by gavage for 7 days and compared weight/organ ratio and evaluated for histomorphology by H&E staining.

NLTC was tested in ICR mice. As the positive control group (Pugazhendhi A. 2018), 2.5 mg/kg/day dose of doxorubicin was injected for 10 days in a row (Yarana et al., 2018). The low-dose group of NLTC (20 g/7.8 mg/day) and high-dose group of NLTC (20 g/39 mg/day) were administered in mice by gavage for 21 days to evaluate whether the drug is toxic and potentially toxic to tissues compared with blank control. The heart rate was monitored using the Langendorff perfusion heart experiment (Langendorff ADI Australia).

Intraperitoneal injection (2.5 mg/kg) of the drug was given continuously for 10 days (Yarana et al., 2018). Blood sample was collected from the animals, and the heart rate was monitored using the Langendorff perfusion heart experiment (Langendorff ADI Australia). Weighing tissue from the internal organs of the mouse (heart, kidney, and liver) were fixed in 4% paraformaldehyde (PFA) solution for over 24 h and further prepared for paraffin sectioning. Tissue sections (3 µm thick) were cut and stained with H&E.

### H&E Staining and Histopathological Scoring

The heart, liver, and kidney tissues were isolated from the mice and weighed and fixed in 4% PFA solution for well over 24 h. Thereafter, specimens were dehydrated through 70−100% ethanol alcohol and cleared in several changes of xylene and embedded in paraffin wax. Transverse serial sections were cut at 3 µm and mounted on glass slides. Sections were stained with H&E. Stained slides were scored according to the degree of organ damage following the reference system by “New Drug Toxicology Experimental Animal Histopathology Map” (Southeast University Press). Evaluation criteria are as shown in **Supplemental Tables 3–5**. Organ coefficient, a common toxicological indicator, was calculated as: organ coefficient = organ weight/body weight.

### Blood Serum Samples Treatment

Serum was obtained from the peripheral blood of ICR mice by centrifugation at 3,000 rpm for 10 min. Level of ALT, AST, LDH, CK-MB, and Scr in the serum were determined by automatic biochemical detector (MK3; Thermo Fisher Scientific, Waltham, MA, USA). Based on the instructions, commercial ELISA kits (R&D Systems, Minneapolis, MN) were used to measure the concentration.

### Cardiac, Renal, and Hepatic Cell Culture

H9c2 (cardiac cells), HEK293 (renal cells), and HepG2 (hepatic cells) were purchased from the Cell Bank of the Chinese Academy of Sciences (Shanghai, China). Cells were cultured in DMEM containing 4.5 g/l glucose, supplemented with 10% fetal bovine serum (FBS) and 1% penicillin/streptomycin. All the cultured cells were maintained in a humidified incubator with 95% air and 5% CO_2_ at 37°C. The cell culture medium was replaced every 2–3 days, and the cells were subcultured or subjected to experimental procedures at 80–90% confluence.

### Cell Viability Assay

Cells were cultured in 96-well plates for 24 h. The seed plate density of H9c2, HepG_2_, and HEK293 cells was 6 × 10^4^/ml, 1 × 10^5^/ml, and 1 × 10^5^/ml, respectively, at 100 µl/well. They were incubated for 24 h at 37°C in a 5% CO_2_ incubator. In 96-well black transvaginal cell culture plates, HepG2 cells and HEK293 cells were each added with 50 µl of 0.025 mg/ml rat tail type I collagen per well overnight, and washed twice with sterile pure water.

The original medium was discarded in the culture plate, and the drug was added at a concentration as a dose standard for examining the effects of nine drugs on the viability of three cell lines, and was placed in a 37°C, 5% CO_2_ incubator and cultured for 24 h.

The cell parameters were measured by Hoechst 33342, which were obtained from Invitrogen (Eugene, USA). After 24-h incubation, 3 μmol/l Hoechst 33342 was mixed with DMEM/high glucose and then incubated with the cells for 30 min in the dark. The assay plate was imaged and analyzed using the Operetta HCA system (Perkin Elmer, MA, USA) at 25°C with a relative humidity of 45%. Using a 20× objective, fluorescent images of nine fields per well in Hoechst 33342 were measured and calculated by the mean values.

### HPLC Analysis

Liquid chromatography was performed on the filtrate using a SunFire-C18 column (4.6 × 250 mm, 5 µm). An isocratic elution program was conducted for chromatographic separation, with the mobile phase A (0.05% KH_2_PO_4_) and mobile phase B (acetonitrile), which was employed using a mixture of 0.05% KH_2_PO_4_ and acetonitrile (75:25, v/v) at a flowrate of 1.0 ml/min (Li et al., 2010). The column temperature was maintained at 30°C, the detection wavelength was 261 nm, and the injection volume was 10 µl for the detection of tolperisone hydrochloride. Under the same conditions, the detection wavelength was changed to 283 nm, and 10 µl was injected for the detection of methyl hesperidin monomer. Liquid chromatography with a UV detector was fixed at a wavelength of 290 nm (Chi and Yin, 2008). Isocratic elution was employed using a mixture of methanol and 0.06% sodium heptane sulfonate (C_7_H_15_NaO_3_S) aqueous solution (13:87, v/v) at a flowrate of 1.0 ml/min, with injection volume of 20 µl for the vitamin B6 analysis.

### Statistical Analysis

All values are expressed as the mean ± SD. Comparisons between multiple-group means were performed using one-way analysis of variance (one-way ANOVA). Multiple comparisons between the groups were performed using least significant difference method. *P* values < 0.05 were considered to be statistically significant. All graphs are drawn by GraphPad Software (GraphPad Software, Inc. CA, USA).

## Results

### *In Vivo* Comparative Toxicology Evaluation of BASR-pCHMs

**Figure 1:**
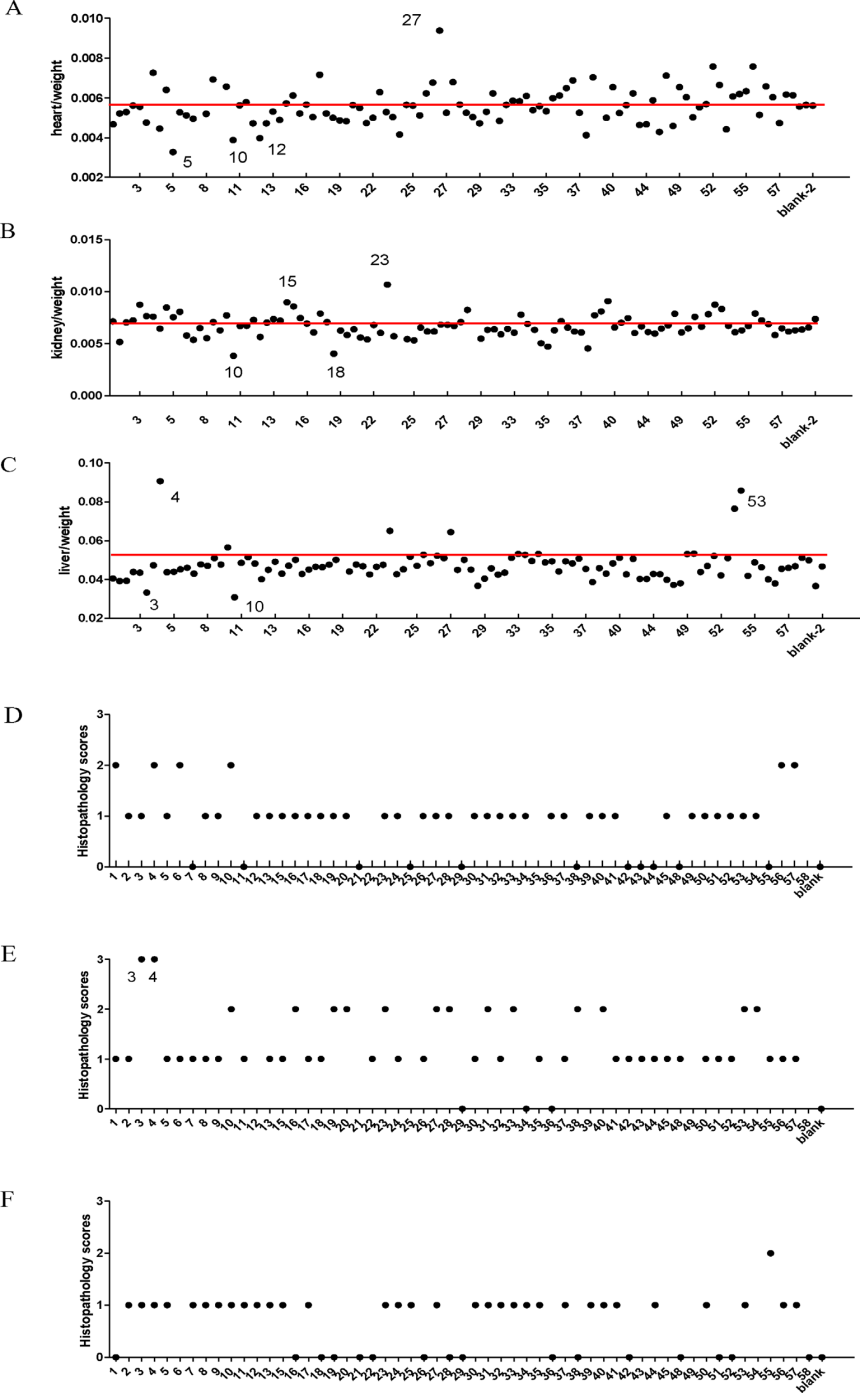
Comparison of organ coefficient and histology scores of BASR-pCHMs-administered mice. **(A**–**C)** Organ/body weight ratio. Redline is the average level. **(A)** Heart/body weight ratio. **(B)** The kidney/body weight ratio. **(C)** The liver/body weight ratio. **(D)** Heart H&E pathology scores. **(E)** Kidney H&E pathology scores. **(F)** Liver H&E pathology scores.

In order to establish a safety pharmacology standard for BASR-pCHMs, we first collected all 58 pCHMs with BASR function and evaluated them *in vivo*. Each of the 58 BASR-pCHMs were given daily by intragastric administration to VEGFR2-Luc mice for 7 days, and duplicate mice were used for each drug. Body and organ weights of the VEGFR-2-Luc mice in each group were measured. As shown in **Figure 1**, the ratios of body weight to heart (**Figure 1A**), kidney (**Figure 1B**), and liver (**Figure 1C**) in most animals treated with BASR-pCHMs were normal. However, the body/heart ratio of animals treated with drugs 5, 10, and 12 were significantly lower. On the other hand, the body/heart ratio of animals treated with drugs 27, 52, and 56 were much higher (**Figure 1A**). In addition, the body/kidney ratio of animals treated with drugs 10 and 18 were significantly lower and that of 23 was higher. Finally, the body/liver ratio of animals treated with drugs 3 and 10 were lower and those of drugs 4, 23, 27, and 53 were higher. H&E tissue stain of BASR-pCHM-treated animals was examined for possible pathological changes. As shown in **Figures 1D–F**, according to a pathological score criteria of 0–3 grades (detailed information in **Supplemental Tables 3–5**), most of the heart (**Figure 1D**), kidney (**Figure 1E**), and liver (**Figure 1F**) tissue stains were normal, except those that were treated with drugs 3 and 4 in kidney. Therefore, a combination of the body/organ ratio and H&E score results identified that treatment by drugs 3, 4, 5, 10, 12, 15, 18, 23, and 27 may cause potential pathological abnormality.

### *In Vitro* Toxicity Evaluation in Cardiac, Renal, and Hepatic Cells of the BASR-pCHMs With Organ Damage Potential

**Figure 2:**
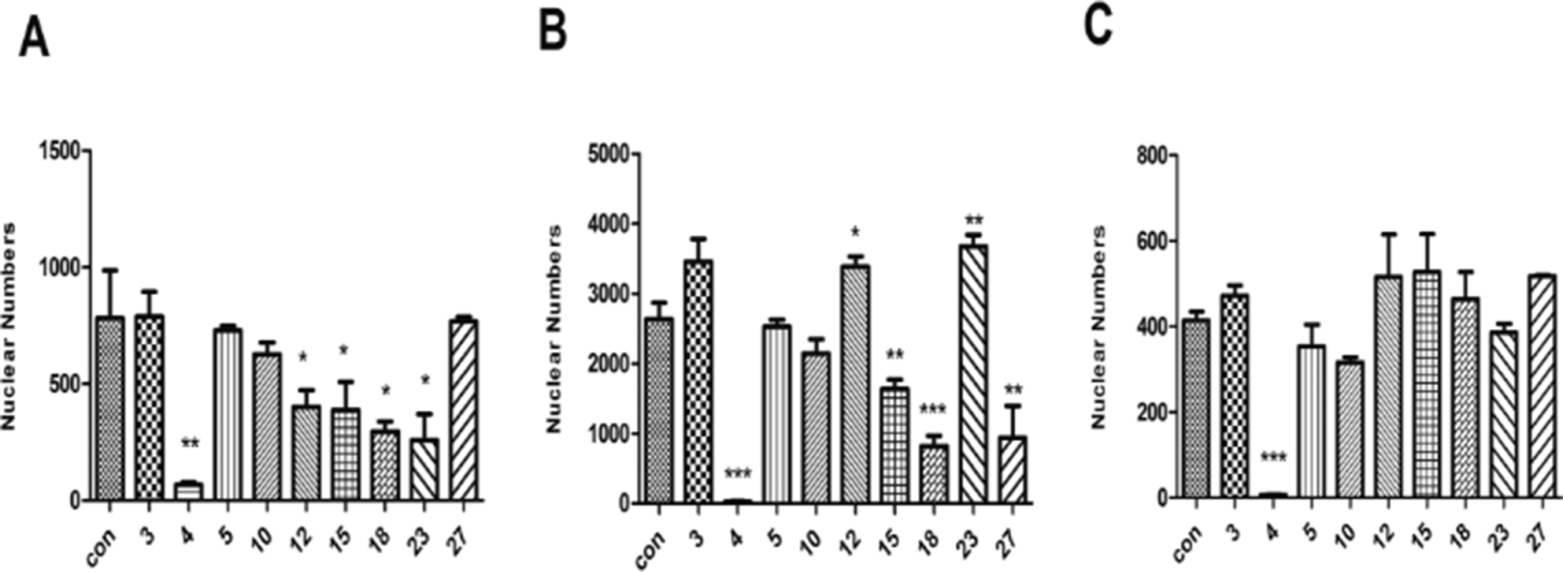
Effects of the nine BASR-pCHMs on the viability of cardiac, renal, and hepatic cells. Hochest-stained cell nuclei were counted after 24 h treatment with the nine BASR-pCHM selected by *in vivo* toxicity screen. **(A)** H9c2 cells. **(B)** HEK293 cells. **(C)** HepG2 cells. Numbers on the *X*-axis are the BASR-pCHM code numbers. Data are expressed as mean ± SD, *n* = 3. *P < 0.05, **P < 0.01, ***P < 0.001 compared with control.

Based on the primary evaluation of the *in vivo* toxicity, drugs 3, 4, 5, 10, 12, 15, 18, 23, and 27 were selected for further investigation in *in vitro* cell models. H9c2, HepG2, and HEK293 cells were chosen to examine the effect of a given drug at a concentration of 1 mg/ml (a concentration that was five times of the maximum effective dose of a compound Chinese medicine in previous *in vitro* study in our laboratory) on cell viability. As shown in **Figure 2**, nuclear numbers of H9c2 (**Figure 2A**), HEK293 (**Figure 2B**), and HepG2 (**Figure 2C**) changed in various fashions after treatment by these nine drugs, but only drug 4 (Naoluotong capsule, NLTC) showed a most dramatic decrease in all of the cardiac, renal, and hepatic cells.

### Composition Analysis of NLTC

**Figure 3:**
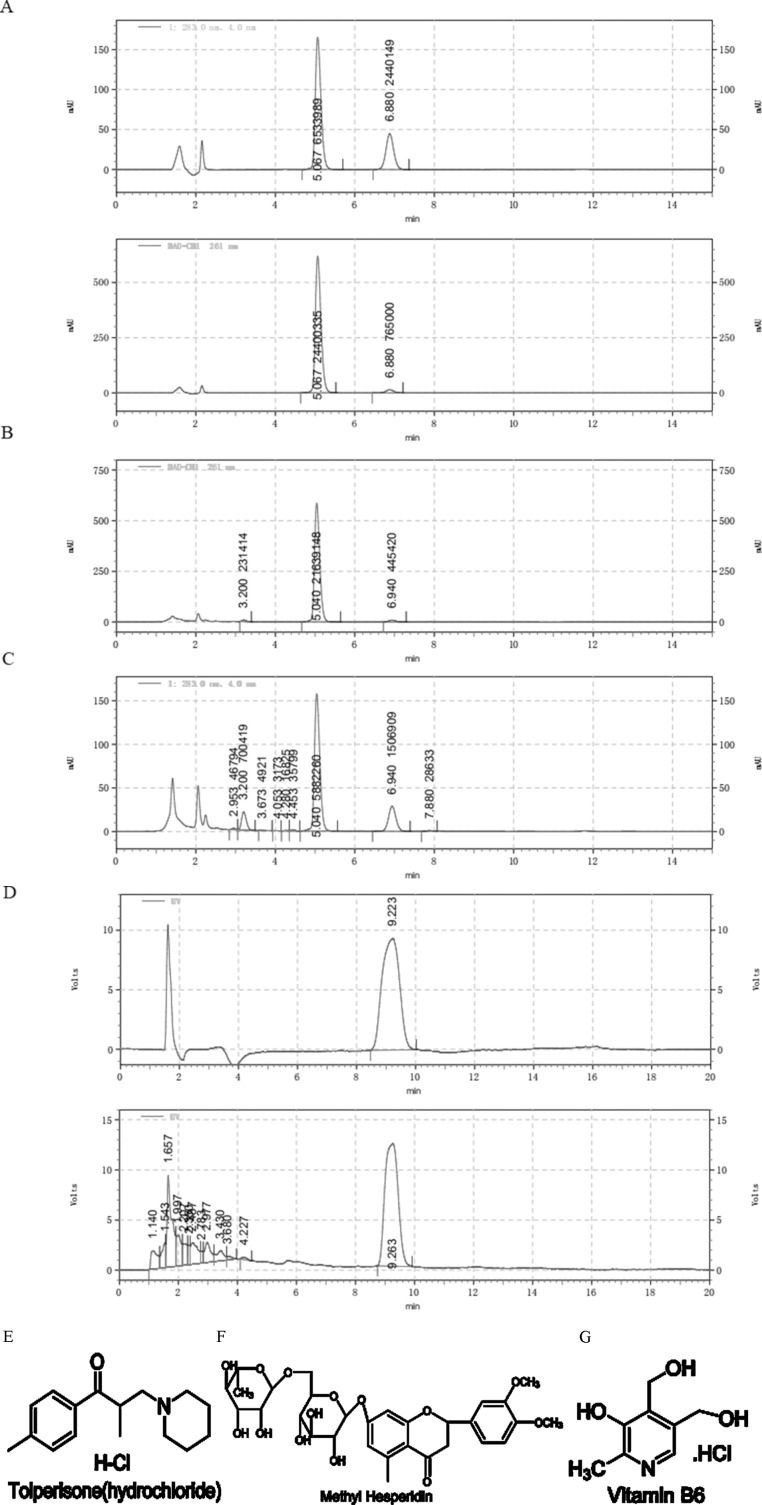
Composition analysis of NLTC. **(A)** Aqueous solution of mixed standards (methyl hesperidin and tolperisone hydrochloride) was detected at 283 nm (upper panel) and 261 nm (lower panel), respectively. **(B)** Aqueous solution of NLTC was detected at wavelength of 261 nm. **(C)** Aqueous solution of NLTC was detected at wavelength of 283 nm. **(D)** Vitamin B6 standard (upper panel) and aqueous solution of NLTC (lower panel) were detected at wavelength of 290 nm. **(E–G)** Structures of the chemical synthetic drug components in NLTC. **(E)** Tolperisone hydrochloride. **(F)** Methyl hesperidin. **(G)** Vitamin B6.

To trace the possible toxic components, composition of the 58 BASR-pCHMs were examined according to the Chinese Pharmacopoeia (10^th^ edition) and the manufacture’s documentations. NTLC was found to be only one of the two BASR-pCHMs that contained chemical synthetic drugs in addition to herbal components. NLTC consists of three CHMs (*Salvia miltiorrhiza*, *Ligustrum* chuanxiong, and *Astragalus membranaceus*) and three chemical synthetic drugs [tolperisone hydrochloride (**Figure 3E**), methyl hesperidin (**Figure 3F**), and vitamin B6 (**Figure 3G**, detailed in **Supplemental Table 6**)]. To determine the contribution of chemical synthetic drugs in NLTC formula, their aqueous extracts were quantified using high-performance liquid chromatography (HPLC) as shown in **Figure 3**. Mixed standards of methyl hesperidin and tolperisone hydrochloride were detected at 283 nm (top) and 261 nm (bottom), respectively (**Figure 3A**), and the presence of tolperisone hydrochloride and methyl hesperidin in NLTC was qualified in **Figures 3B, C**, respectively. As shown in **Figure 3D**, vitamin B6 standard (top) and its presence in NLTC (bottom) were detected at 290 nm. The quantitative results showed that NLTC contained 8.59% per mg/ml tolperisone hydrochloride, 1.20% per mg/ml methyl-hesperidin, and 0.38% per mg/ml vitamin B6, respectively. In addition, the methanol extracts of the chemical synthetic drugs in NLTC were also quantified, and the results were consistent with those of aqueous extracts (**Supplemental Figure 1**).

### Toxicity Effects on Cardiac, Renal, and Hepatic Cells of NLTC Produced by Different Manufacturers

**Figure 4:**
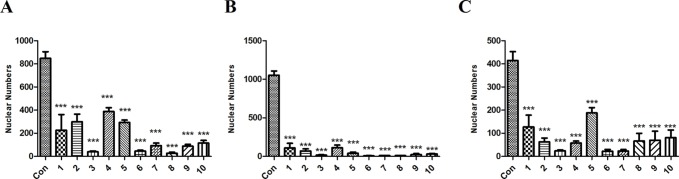
Effects of NLTC produced by different manufacturers on the viability of cardiac, renal, and hepatic cells. Ten different manufacturers of NLTC were represented in the *X*-axis. Con: vehicle control. **(A)** H9c2 cells. **(B)** HEK293 cells. **(C)** HepG2 cells. All drugs were used at a concentration of 1 mg/ml. Data are expressed as mean ± SD, *n* = 3. *P < 0.05, **P < 0.01, ***P < 0.001 compared with control.

To rule out the possibility that the toxic effect by NLTC might have been caused by the preparation process, 10 batches (all at doses of 1 mg/ml) of NLTC from different manufacturers were analyzed by cellular toxicity assay. As shown in **Figure 4**, NLTCs from different manufacturers all showed a significant decrease in nuclear numbers of cardiac (**Figure 4A**), renal (**Figure 4B**), and hepatic (**Figure 4C**) cells, supporting that it was the composition, not the production technique, of NLTC that caused cell toxicity.

### *In Vivo* Verification of NLTC Cellular Toxicity

As shown in **Table 1**, high doses of NLTC and positive control drug Adriamycin significantly elevated serum levels of cardiac function indicators CK-MB and LDH as well as liver function indicators AST and ALT compared with that of the vehicle control, while low dose of NLTC only elevated CK-MB and LDH levels but not AST and ALT levels. In addition, all the drugs significantly reduced the renal function indicator Scr.

**Table 1:** Serum levels of LDH, CK-MB, ALT, AST, and Scr (x¯ ± s, *n* = 3–5).

	LDH (U/l)	CK-MB (U/l)	ALT (U/l)	AST (U/l)	Scr (U/l)
Con	649.75 ± 74.54	267.5 ± 9.49	29.75 ± 2.23	94.5 ± 6.47	38.70 ± 1.54
Dox	699.33 ± 100.52	588 ± 113.19^###^	49.5 ± 3.63	168.75 ± 13.12^##^	34.695 ± 1.18***
NLTC-low	401.4 ± 28.03^###^	432.2 ± 15.32^##^	21.8 ± 1.76	76.8 ± 7.26	30.51 ± 0.47***
NLTC-high	477.2 ± 49.49^##^	370.4 ± 34.36^#^	118.8 ± 22.41^###^	261.2 ± 37.49^###^	27.99 ± 0.79***

Data are expressed as mean *±* SD, n = 3–5. ^#^Represents increased level. ^#^P < 0.05, ^##^ P < 0.01, ^###^ P < 0.001 compared with control. *represents decreased level. ***P < 0.001 compared with control.

**Figure 5:**
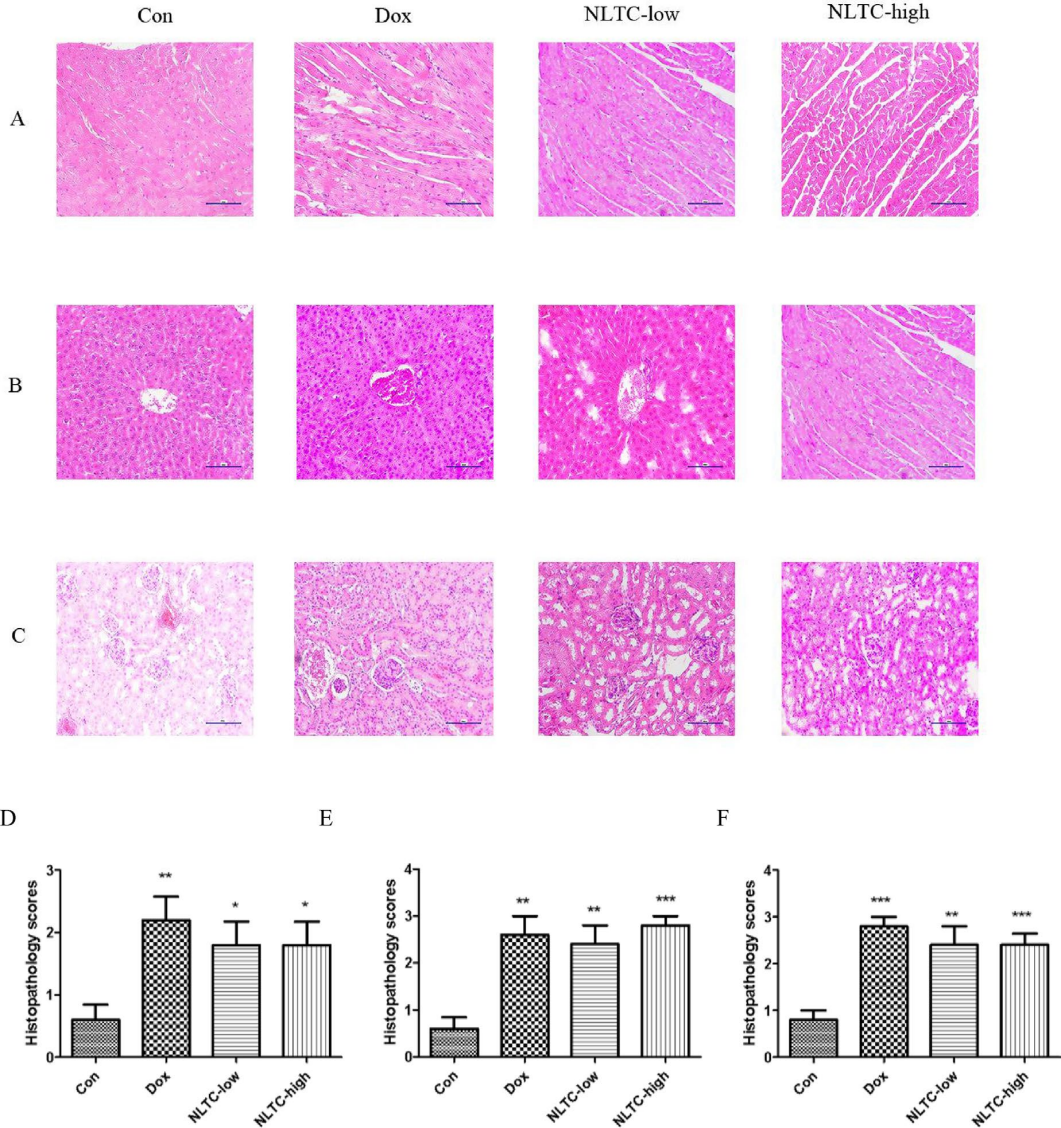
Histopathology of NLTC-treated animals experimental scores. **(A–C)** Representative images of H&E-stained tissues. **(A)** cardiac tissue. **(B)** hepatic tissue. **(C)** Renal tissue. All images were under 100× microscope. Order of panels from left to right: Con (negative control), Dox (positive control), NLTC-low, and NLTC-high. **(D–F)** Quantitation of the H&E histopathology scores. **(D)** cardiac tissue. **(E)** Hepatic tissue. **(F)** Renal tissue. Data are expressed as mean ± SD, *n* = 5. *P < 0.05, **P < 0.01, ***P < 0.001 compared with control.

As shown in **Figure 5**, the morphology of H&E-stained heart (**Figure 5A**), liver (**Figure 5B**), and kidney (**Figure 5C**) indicated typical pathological changes. Quantitation of the H&E stain showed that NLTC significantly increased pathological scores in the heart (**Figure 5D**), liver (**Figure 5E**), and kidney (**Figure 5F**) compared with the control.

### Toxicity Mechanisms of NLTC and Its Chemical Synthetic Drug Combination

**Figure 6:**
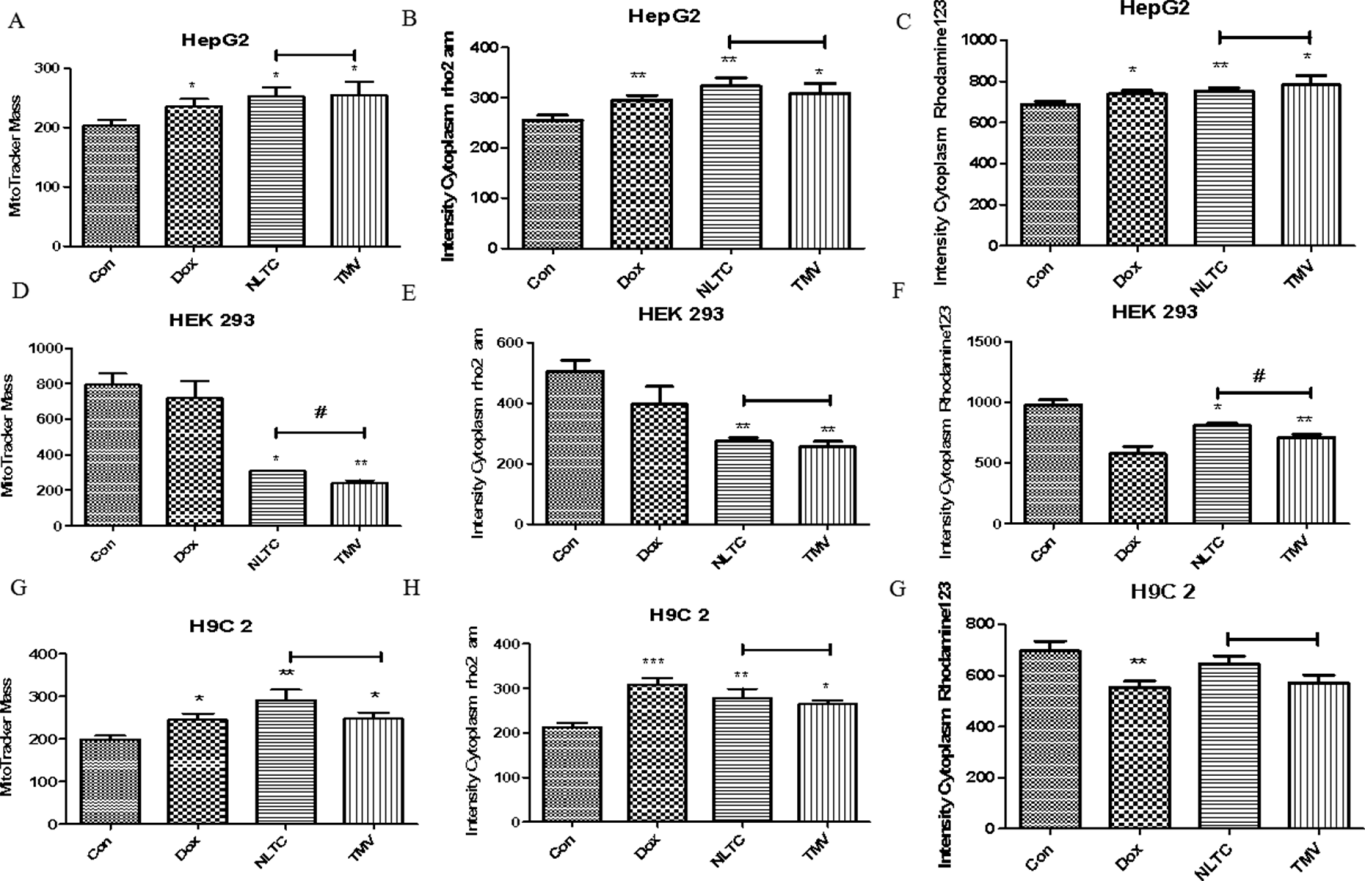
Toxicity mechanisms of NLTC and its chemical synthetic drug combination. Multiparameter cell-based imaging analysis was performed after 24 h drug treatment. Mitochondrial mass, intracellular calcium, and mitochondrial membrane potential were determined in cardiac, renal, and hepatic cells. **(A, D, G)** Mitochondrial mass. **(B, E, H)** Intracellular calcium. **(C, F, I)** Mitochondrial membrane potential. **(A–C)** HepG2 cells. **(D–F)** HEK293 cells. **(G–I)** H9c2 cells. Con: control, Dox: positive control, NLTC (NLTC aqueous extract), TMV: mixtures of tolperisone hydrochloride, methyl hesperidin, and vitamin B6. *P < 0.05, **P < 0.01 compared to the control. ^#^ *P* < 0.05 NLTC compared to TMV.

In order to further clarify the contribution of the chemical synthetic drug combination to the cytotoxicity of total NLTC and their toxicity mechanisms, the three chemical synthetic drugs (tolperisone hydrochloride, methyl hesperidin, and vitamin B6, TMV) were formulated according to the same proportion of their presence in NLTC. In a cell-based multiparameter toxicity assay, aqueous extract of NLTC and its chemical synthetic drug combination TMV were examined as shown in **Figure 6**. Mitochondrial mass (**Figures 6A, D, G**), calcium ion concentration (**Figures 6B, E, H**), and mitochondrial membrane potential (**Figures 6C, F, I**) were determined in HepG2 (**Figures 6A–C**), HEK293 (**Figures 6D–F**), and H9c2 (**Figures 6G–I**) cells. Similar to the positive control drug DOX, both NLTC and TMV affected mitochondrial mass, calcium ion concentration, and mitochondrial membrane potential in the same fashion in most cells, except for the renal HEK293 cells where mitochondrial functional parameters were more severely affected by TMV than NLTC (**Figures 6D, F**).

### Attribution of Individual Chemical Synthetic Drugs From NLTC on Cellular Toxicity

**Figure 7:**
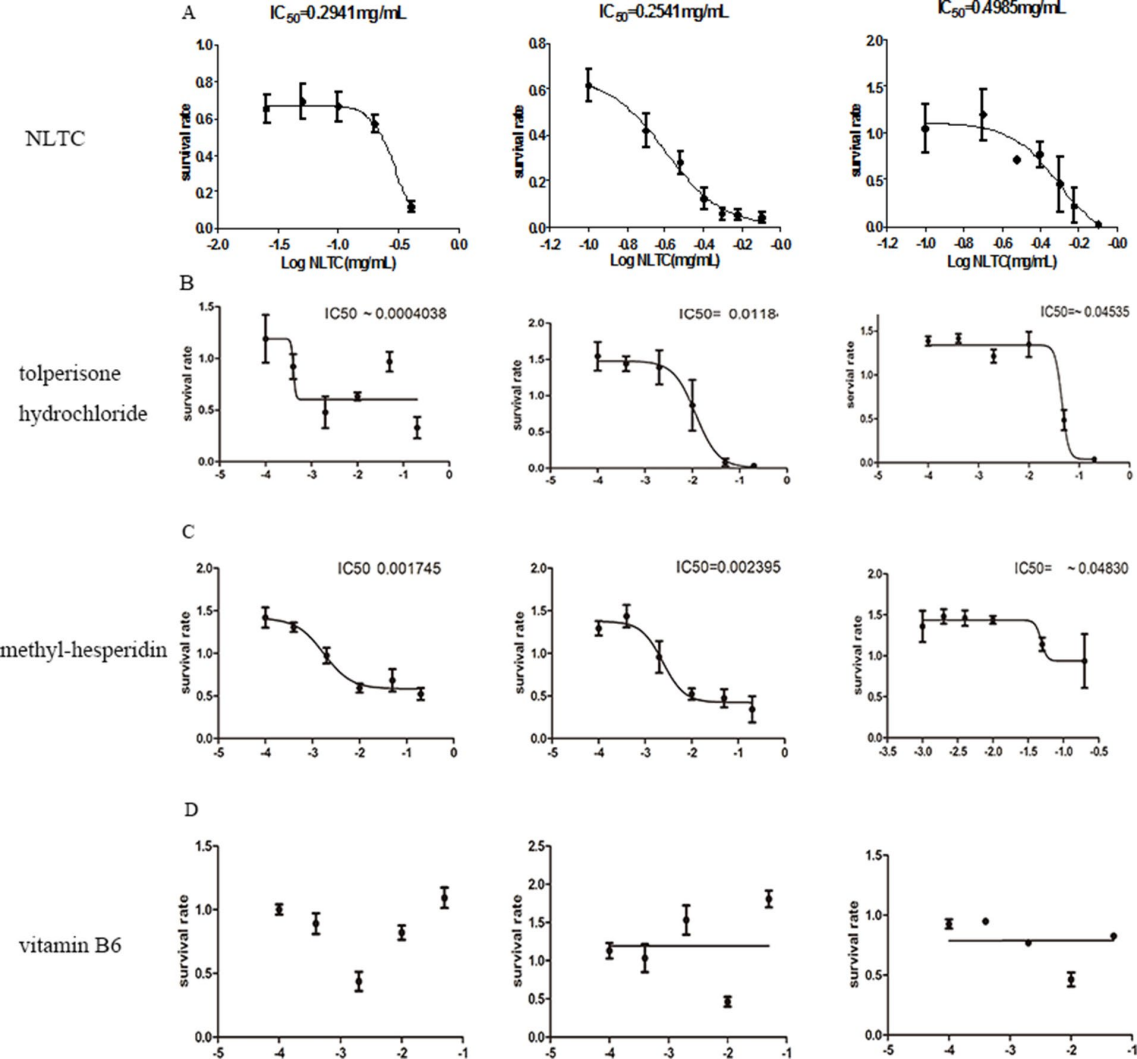
Attribution of individual chemical synthetic drugs from NLTC on cellular toxicity. Survival rate of cells after 24 h treatment by NLTC **(A)**, tolperisone hydrochloride **(B)**, methyl hesperidin **(C)**, and vitamin B6 **(D)**. Left panels: H9c2; middle panels: HepG2; right panels: HEK293. Values of IC50, where available, are indicated in the panel. Data are expressed as mean ± SEM, *n* = 9.

Cellular toxicity by tolperisone hydrochloride, methyl-hesperidin, and vitamin B6, the three chemical synthetic drugs in NLTC, was analyzed in cardiac, hepatocyte, and renal cell lines. As shown in **Figure 7**, compared with that of NLTC (**Figure 7A**), which had IC50s on H9c2 cell, HepG2, and HEK293 cells of 0.2941, 0.02541, and 0.4985 mg/ml, tolperisone hydrochloride (**Figure 7B**) significantly caused a lethal effect on H9c2 cell, HepG2, and HEK293 cells with IC50 of 0.004038, 0.0118, and 0.04535 mg/ml, respectively. Methyl-hesperidin (**Figure 7C**) also significantly caused a lethal effect on H9c2 cell, HepG2, and HEK293 cells with IC50 of 0.001745, 0.002395, and 0.04830 mg/ml, respectively. However, vitamin B6 (**Figure 7D**) had no effect on these cells. Therefore, the cellular toxicity of NLTC could be attributed mostly to tolperisone hydrochloride, but to a less extent, methyl-hesperidin.

## Discussion

Safety reevaluation of patent Chinese herbal medicine already on market is challenging due to their complex chemical composition and ambiguous clinical indications. To adapt the adverse outcome pathway (AOP) approach for pCHM, 58 BASR-pCHMs were evaluated *in vivo* and *in vitro* screening. New discoveries in this study include 1) untargeted screen revealed that the one BASR-pCHM (NLTC) that may cause organ damage is in fact a mixture of CHM and chemical synthetic drug components; 2) NLTC’s multi cellular toxicity is conformed *in vivo* in a mouse model; and 3) the NLTC chemical synthetic drugs alone, particularly tolperisone hydrochloride, are the cause of cytotoxicity.

Blood activating and stasis removing (BASR) is the main principle in traditional Chinese medicine for treatment of cardiovascular diseases. Previous studies have reported that BASR-CHMs promote angiogenesis by upregulating vascular endothelial growth factor (VEGF) gene in the ischemic model (Xin et al., 2018; Zheng et al., 2018; Shi et al., 2019) and inhibit angiogenesis mainly by downregulating VEGF and decreasing MMP9 expression in tumor tissues (Zeng et al., 2018). There are also reports in the literature of the side effect caused by BASRs in abnormally promoting normal blood vessel growth (Su et al., 2014). We used VEGFR2-luc reporter mice to explore the effect of BASR-pCHMs on angiogenesis *in vivo* and possible correlation of tissue-specific VEGF gene expression and organ response. Bioluminescence results of luciferase activity showed occasional VEGF elevation induced by certain BASR-pCHM, but it was not related to the toxicity effects reported in this work (data not shown).

According to AOP approach, we integrated animal, cellular, and molecular parameters for the BASR-pCHMs evaluation. The animal level screen of organ toxicity was informative but did not reach statistical significance. Since the organ weight changes may or may not be directly affected by BASR-pCHMs, we considered histopathology score as additional evaluation parameter.

Although NLTC has been used as a cerebral vascular disease treatment medicine in China, its safety has not been systematically examined except occasional reports of adverse reactions such as allergies (Liu et al., 2004; Kang et al., 2017; Bai et al., 2019). Our experimental study suggested that NLTC could potentially cause multiorgan toxicity.

The results of H&E stain in NLTC animal experiments are inconsistent with serum index results in our study. H&E stain was used to evaluate the changes in tissues from mice with different treatments. The results showed that the volume of the myocardium in the doxorubicin (Dox) group was smaller than that in the control group and the CK-MB and LDH levels increased by Dox. This is consistent with the literature report (Deng et al., 2018). In Dox-induced nephrotoxicity animal experiment, renal sections from Dox-treated rats showed intensive desquamation in tubular epithelial cells, single-cell necrosis, tubular atrophy, tubular necrosis, and glomerular necrosis (Altinkaynak et al., 2018). According to the Kidney Disease: Improving Global Outcomes (KDIGO) guidelines, acute kidney injury is defined as an increase in the serum creatinine level of 0.3 mg/dl or greater within 48 h or an increase in serum creatinine levels to 1.5 or greater times the baseline (Joyce et al., 2017). However, the level of renal function parameters of NLTC-treated animals was signiﬁcantly lower compared to that of control in our results. The results show that the drug damage to the kidney was nonacute injury, but the specific type is affected by many factors and needs further analysis (Takeda et al., 2018). Dox-induced hepatotoxicity significantly increases the levels of ALT and AST in serum (Wang et al., 2014). In our result, serum ALT and AST levels were normal in the low dose of NLTC-treated animals. However, levels of ALT and AST in serum were significantly increased in the high dose of NLTC-treated animals. Therefore, our data suggest that the drug dose is a critical factor for the safe clinical application of NLTC, and potential organ damage depends on the sensitivity of different organs (i.e., kidney vs. liver). However, our experimental data should be interpreted with caution, and a detailed clinical investigation is needed to confirm if NLTC indeed may cause multiorgan toxicity in patients.

The toxicity of toperidone hydrochloride, methyl hesperidin, and vitamin B6 has not been reported before. The side effects of tolperisone hydrochloride reported at present is not clear (Ribi et al., 2003; Sporkert et al., 2012; Martos et al., 2015). In animal studies, methyl hesperidin has not been found to have toxic or side effects before (Kawabe et al., 1993). As for vitamin B6, previous reports have shown that its combination with other drugs may reduce the toxicity or side effects of the later (Wang L.Z. et al., 2015). However, our work suggested that tolperisone hydrochloride may cause cellular toxicity *in vitro*, although this effect has yet to be confirmed *in vivo*.

Dox is a well-established anticancer drug, but it broad-spectrum cytotoxicity has also been reported (Nishiyama et al., 2019). Using Dox as a cellular toxicity positive control, we determined each of the chemical synthetic drugs in NLTC for the mechanistic insights, such as mitochondrial mass, mitochondrial membrane potential, and intracellular calcium. The results showed that the administration of the combined chemical synthetic drugs or NLTC aqueous extract exhibited similar effects as Dox in cardiac, renal, and hepatic cells, suggesting that these drugs may share the same toxicity mechanism(s). Our results showed that in cardiomyocytes and hepatocytes, the toxicity levels of the combined chemical synthetic drugs and NLTC aqueous extract were similar, whereas in the renal cells, the toxicity level of the combined chemical synthetic drugs was more significant than that of NLTC. It may suggest that kidney is a more sensitive organ for chemical toxicity than the heart and liver. Alternatively, it is possible that the presence of the herbal components decreased the chemical toxicity in kidney by drug–drug interaction.

One of the limitations of our work is that the chemical synthetic drug components in NLTC were verified only at cellular level, and no *in vivo* animal experiments were carried out. In addition, we have not ruled out the possibility of drug–drug interaction between the components of chemical synthetic drugs and herbs. In addition, although our cell-based multiparameter assays revealed preliminary mechanisms, further in-depth investigation is required to fully understand if and how NLTC might lead to multiorgan toxicity in clinics.

For the first time, this study evaluated the safety of a series of commercially available pCHMs, from the levels of the whole animal, selected tissues, and targeted cells. Compared with conventional toxicity studies that focus a single CHM formula or a monomer, our approach offers a systematic evaluation which allows an unbiased comparison of multiple drugs.

In conclusion, our study for the first time systematically evaluated a series of patent herbal medicines using an integrated approach and identified the presence of chemical synthetic drug in an herbal medicine as a potential cause of organ toxicity.

## Ethics Statement

All animal experiments were performed in accord with the international regulations, following the guidelines of Tianjin University of TCM Animal Research Committee (TCM-LAEC2014004) and approved by the animal care and use committee of TJAB (No. TJU20160021).

## Author Contributions

YZ conceived the project and designed the study. XL performed the experiments, contributed to acquisition, analysis, and interpretation of the data. RS supervised the experiment and revised the manuscript. XY, GX, and YF contributed to study design and interpretation of results. XL, RS, and YZ wrote the manuscript. All authors reviewed and approved the manuscript.

## Funding

This study was supported by the grants from National Science Foundation of China (81274128, 81873037), Major National Science and Technology Projects (2018YFC1704500), and Innovation Team of Tianjin High Education Commission (TD13-5046).

## Conflict of Interest Statement

The authors declare that the research was conducted in the absence of any commercial or financial relationships that could be construed as a potential conflict of interest.

## Abbreviations

BASR-pCHM, blood-activating and stasis-removing patent Chinese herbal medicine; NLTC, Naoluotong capsule; CHM, Chinese herbal medicine; pCHM, patent Chinese herbal medicine; LDH, lactate dehydrogenase; AST, aspartate aminotransferase; CK-MB, creatine kinase-MB; Scr, serum creatinine; H&E, hematoxylin and eosin.
