# Supplementary material for: Untargeted Safety Pharmacology Screen of Blood-Activating and Stasis-Removing Patent Chinese Herbal Medicines Identified Nonherbal Ingredients as a Cause of Organ Damage in Experimental Models
Source: Front Pharmacol. 2019 Sep 12;10:993. doi: 10.3389/fphar.2019.00993 (PMC6757273; doi:10.3389/fphar.2019.00993)
Supplement: Supplementary file 2 [file DataSheet_2.docx]

Table1: The information of BASRMs

| The name of  BASRMs | Main components | batch number | Chinese herbal medicinal ingredient number | Chemical monomer  ingredient number | Chemical composition of the whole drug proportion |
| --- | --- | --- | --- | --- | --- |
| 1. shensongyangxin capsule 2. kangerxin capsule 3. kangshuan capsule 4. naoluotong capsule 5. linaoxin capsule 6. qiliqiangxin capsule 7. tiandantongluo capsule 8. tongxinluo capsule 9. xinkening capsule 10. xinkeshu capsule 11. xinnaokang capsule 12. xuefuzhuyu capsule 13. Buchangnaoxintongcapsule 14. danqi [tablet](D:/Youdao/Dict/8.3.1.0/resultui/html/index.html#/javascript:;) 15. fufangdanshen [tablet](D:/Youdao/Dict/8.3.1.0/resultui/html/index.html#/javascript:;)（beijing） 16. fufangdanshen [tablet](D:/Youdao/Dict/8.3.1.0/resultui/html/index.html#/javascript:;)（yunnan） 17. guanmaining  [tablet](D:/Youdao/Dict/8.3.1.0/resultui/html/index.html#/javascript:;) 18. huoxuetongmai [tablet](D:/Youdao/Dict/8.3.1.0/resultui/html/index.html#/javascript:;) 19. jingzhiguanxin [tablet](D:/Youdao/Dict/8.3.1.0/resultui/html/index.html#/javascript:;) 20. naodesheng  [tablet](D:/Youdao/Dict/8.3.1.0/resultui/html/index.html#/javascript:;) 21. naoxueshuan  [tablet](D:/Youdao/Dict/8.3.1.0/resultui/html/index.html#/javascript:;) 22. xiaoshuantongluo  [tablet](D:/Youdao/Dict/8.3.1.0/resultui/html/index.html#/javascript:;) 23. xinkeshu  [tablet](D:/Youdao/Dict/8.3.1.0/resultui/html/index.html#/javascript:;) 24. yangxinshi  [tablet](D:/Youdao/Dict/8.3.1.0/resultui/html/index.html#/javascript:;) 25. zhijiangning  [tablet](D:/Youdao/Dict/8.3.1.0/resultui/html/index.html#/javascript:;) 26. Guanmaitong  [tablet](D:/Youdao/Dict/8.3.1.0/resultui/html/index.html#/javascript:;) 27. Fufangdanshend pill 28. qishenyiqidi pill 29. Huatuozaizao pill 30. huoxin pill 31. renshenzaizao pill 32. xiaoshuanzaizao pill 33. xuefuzhuyu pill 34. lemai granules 35. tongmai granules 36. xuefuzhuyu granules 37. wenxin granules 38. tongmaiyangxin pill 39. dengzhanshengmai capsule 40. xinbao pill 41. zhuyutongmai capsule 42. hedan tablet 43. tongmaijiangzhi tablet 44. xuefuzhuyu tablet 45. naoan capsule 46. yixin pill 47. xueshuanxinmaining capsule 48. zhengxintai capsule 49. Danxiongtongmai granules 50. Fufangxueshuantong capsule 51. Danhong [injection](D:/Youdao/Dict/8.3.1.0/resultui/html/index.html#/javascript:;) 52. Xiangdan [injection](D:/Youdao/Dict/8.3.1.0/resultui/html/index.html#/javascript:;) 53. Shuxuetong [injection](D:/Youdao/Dict/8.3.1.0/resultui/html/index.html#/javascript:;) 54. Xingnaojing [injection](D:/Youdao/Dict/8.3.1.0/resultui/html/index.html#/javascript:;) 55. Mailuoning [injection](D:/Youdao/Dict/8.3.1.0/resultui/html/index.html#/javascript:;) 56. Xuebijing [injection](D:/Youdao/Dict/8.3.1.0/resultui/html/index.html#/javascript:;) 57. Shenmai [injection](D:/Youdao/Dict/8.3.1.0/resultui/html/index.html#/javascript:;) 58. Shenfu [injection](D:/Youdao/Dict/8.3.1.0/resultui/html/index.html#/javascript:;) | [panax ginseng](D:/Youdao/Dict/8.3.1.0/resultui/html/index.html#/javascript:;),  Radix Ophiopogonis, etc  Panax notoginseng, ginseng, etc  Angelica tail, salvia miltiorrhiza, etc  Salvia miltiorrhiza ,  Ligusticum wallichii, etc  Salvia miltiorrhiza ,  Ligusticum wallichii, etc  Astragalus membranaceus,Ligusticum wallichii, etc  Salvia miltiorrhiza ,  Ligusticum wallichii, etc  [panax ginseng](D:/Youdao/Dict/8.3.1.0/resultui/html/index.html#/javascript:;),leech, etc  Salvia miltiorrhiza, panax notoginseng,etc  Salviamiltiorrhiza, Pueraria etc  Radix paeoniae rubra,Salvia miltiorrhiza,etc  peach kernel,flos carthami,etc  radix astragali ,Radix Paeoniae Rubra,etc  salvia miltiorrhiza,Panax notoginseng  salvia miltiorrhiza,Panax notoginseng,etc  salvia miltiorrhiza,Panax notoginseng,etc  salvia miltiorrhiza,myrrh,etc  Caulis Spatholobi , [Semen Pruni Persicae](D:/Youdao/Dict/8.3.1.0/resultui/html/index.html#/javascript:;), ,etc  Radix paeoniae rubra,  Ligusticum wallichii,etc  Panax notoginseng,Ligusticum wallichii,etc  flos carthami, Chinese Angelica,etc  Ligusticum wallichii,Salvia miltiorrhiza ,etc  Salvia miltiorrhiza ,Pueraria,etc  Astragalus membranaceus,[panax ginseng](D:/Youdao/Dict/8.3.1.0/resultui/html/index.html#/javascript:;),etc  Hawthorn,Radix Polygonum Multiflorum Preparata,etc  wolfberry fruit,polygonum multiflorum,etc  Salvia miltiorrhiza ,Panax notoginseng,etc  Astragalus membranaceus,Salvia miltiorrhiza ,etc  Ligusticum wallichii, evodia rutaecarpa,etc  [panax ginseng](D:/Youdao/Dict/8.3.1.0/resultui/html/index.html#/javascript:;),glossy ganoderma,etc  [panax ginseng](D:/Youdao/Dict/8.3.1.0/resultui/html/index.html#/javascript:;),long-nosed pit viper,etc  daemonorops draco, radix paeoniae rubra,etc  [radix](D:/Youdao/Dict/8.3.1.0/resultui/html/index.html#/javascript:;) [bupleuri](D:/Youdao/Dict/8.3.1.0/resultui/html/index.html#/javascript:;),[Chinese](D:/Youdao/Dict/8.3.1.0/resultui/html/index.html#/javascript:;) [angelica](D:/Youdao/Dict/8.3.1.0/resultui/html/index.html#/javascript:;) ,etc  Salvia miltiorrhiza ,  Ligusticum wallichii, etc  Salvia miltiorrhiza ,  Ligusticum wallichii, etc  [radix](D:/Youdao/Dict/8.3.1.0/resultui/html/index.html#/javascript:;) [bupleuri](D:/Youdao/Dict/8.3.1.0/resultui/html/index.html#/javascript:;),[Chinese](D:/Youdao/Dict/8.3.1.0/resultui/html/index.html#/javascript:;) [angelica](D:/Youdao/Dict/8.3.1.0/resultui/html/index.html#/javascript:;) ,etc  [Codonopsis](D:/Youdao/Dict/8.3.1.0/resultui/html/index.html#/javascript:;) [pilosula](D:/Youdao/Dict/8.3.1.0/resultui/html/index.html#/javascript:;),[rhizoma](D:/Youdao/Dict/8.3.1.0/resultui/html/index.html#/javascript:;) [polygonati](D:/Youdao/Dict/8.3.1.0/resultui/html/index.html#/javascript:;) ,etc  Rehmannia glutinosa,[Suberect](D:/Youdao/Dict/8.3.1.0/resultui/html/index.html#/javascript:;) [Spatholobus](D:/Youdao/Dict/8.3.1.0/resultui/html/index.html#/javascript:;) [Stem](D:/Youdao/Dict/8.3.1.0/resultui/html/index.html#/javascript:;) ,etc  [Erigeron](D:/Youdao/Dict/8.3.1.0/resultui/html/index.html#/javascript:;) [breviscapus](D:/Youdao/Dict/8.3.1.0/resultui/html/index.html#/javascript:;),[panax ginseng](D:/Youdao/Dict/8.3.1.0/resultui/html/index.html#/javascript:;),etc  [flos](D:/Youdao/Dict/8.3.1.0/resultui/html/index.html#/javascript:;) [daturae](D:/Youdao/Dict/8.3.1.0/resultui/html/index.html#/javascript:;),[panax ginseng](D:/Youdao/Dict/8.3.1.0/resultui/html/index.html#/javascript:;),etc leech ,[Semen Pruni Persicae](D:/Youdao/Dict/8.3.1.0/resultui/html/index.html#/javascript:;),etc  folium nelumbinis,Salvia miltiorrhiza ,etc  [Equisetaceae Equisetum debile](D:/Youdao/Dict/8.3.1.0/resultui/html/index.html#/javascript:;),Ligusticum wallichii, etc  [Semen Pruni Persicae](D:/Youdao/Dict/8.3.1.0/resultui/html/index.html#/javascript:;),flos carthami,etc  Ligusticum wallichii, [Chinese](D:/Youdao/Dict/8.3.1.0/resultui/html/index.html#/javascript:;) [angelica](D:/Youdao/Dict/8.3.1.0/resultui/html/index.html#/javascript:;) ,etc  [radix](D:/Youdao/Dict/8.3.1.0/resultui/html/index.html#/javascript:;) [ginseng](D:/Youdao/Dict/8.3.1.0/resultui/html/index.html#/javascript:;) [Rubra](D:/Youdao/Dict/8.3.1.0/resultui/html/index.html#/javascript:;),  [cattle horn](D:/Youdao/Dict/8.3.1.0/resultui/html/index.html#/javascript:;),etc  [Ligusticum](D:/Youdao/Dict/8.3.1.0/resultui/html/index.html#/javascript:;) [wallichii](D:/Youdao/Dict/8.3.1.0/resultui/html/index.html#/javascript:;) ,[Flos](D:/Youdao/Dict/8.3.1.0/resultui/html/index.html#/javascript:;) [Sophorae](D:/Youdao/Dict/8.3.1.0/resultui/html/index.html#/javascript:;) ,etc  Astragalus membranaceus,Pueraria,etc  Salvia miltiorrhiza ,  flos carthami,etc  Astragalus membranaceus,Panax notoginseng,etc  Salvia miltiorrhiza ,[flos carthami](D:/Youdao/Dict/8.3.1.0/resultui/html/index.html#/javascript:;),etc  Salvia miltiorrhiza ,Lignum Dalbergiae Odoriferae,  Leech,[Earthworm](D:/Youdao/Dict/8.3.1.0/resultui/html/index.html#/javascript:;)  [musk](D:/Youdao/Dict/8.3.1.0/resultui/html/index.html#/javascript:;),[borneol](D:/Youdao/Dict/8.3.1.0/resultui/html/index.html#/javascript:;) ,etc  [Lonicera](D:/Youdao/Dict/8.3.1.0/resultui/html/index.html#/javascript:;) [japonica](D:/Youdao/Dict/8.3.1.0/resultui/html/index.html#/javascript:;),[radix](D:/Youdao/Dict/8.3.1.0/resultui/html/index.html#/javascript:;) [achyranthis](D:/Youdao/Dict/8.3.1.0/resultui/html/index.html#/javascript:;) [bidentatae](D:/Youdao/Dict/8.3.1.0/resultui/html/index.html#/javascript:;),etc  [flowers](D:/Youdao/Dict/8.3.1.0/resultui/html/index.html#/javascript:;) [carthami](D:/Youdao/Dict/8.3.1.0/resultui/html/index.html#/javascript:;) ,  [Radix Paeoniae Rubra](D:/Youdao/Dict/8.3.1.0/resultui/html/index.html#/javascript:;),etc  [radix](D:/Youdao/Dict/8.3.1.0/resultui/html/index.html#/javascript:;) [ginseng](D:/Youdao/Dict/8.3.1.0/resultui/html/index.html#/javascript:;) [Rubra](D:/Youdao/Dict/8.3.1.0/resultui/html/index.html#/javascript:;) ,[Radix](D:/Youdao/Dict/8.3.1.0/resultui/html/index.html#/javascript:;) [Ophiopogonis](D:/Youdao/Dict/8.3.1.0/resultui/html/index.html#/javascript:;)  [radix](D:/Youdao/Dict/8.3.1.0/resultui/html/index.html#/javascript:;) [ginseng](D:/Youdao/Dict/8.3.1.0/resultui/html/index.html#/javascript:;) [Rubra](D:/Youdao/Dict/8.3.1.0/resultui/html/index.html#/javascript:;) ,sliced aconite | 1108021  20110311  110901  120101  1110002  111202  1109224  110939  110601  110202  20120104  B03020  111186  B89001  12126606  20110477  20120202  120367  211002  20120312  20111101  1124634  110606  110616  120103  82001  111123  120509  1426  1120001  111201  1013528  120413  120114  111110  110926  120821  B107253  20120905  20120501  20121002  20121006  20120401  120801  120101  110905  120910  20120701  1111002  120821  13081025  20120404  11111122  1112091  20111006  1207161  1107201  110804 | 12  7  12  3  15  11  10  12  8  5  10  11  16  2  3  3  14  17  5  5  10  11  5  13  8  7  3  4  7  10  55  38  11  7  3  11  5  11  4  9  4  5  5  11  5  11  10  6  8  4  2  2  2  4  4  2  2  5 | 0  0  0  3  0  0  0  0  0  0  0  0  0  0  0  0  0  0  0  0  0  0  0  0  2  0  0  0  0  0  0  0  0  0  0  0  0  0  0  0  0  0  0  0  0  0  0  0  0  0  0  0  0  0  0  0  0  0 | 0  0  0  12.4%  0  0  0  0  0  0  0  0  0  0  0  0  0  0  0  0  0  0  0  0  2.56%  0  0  0  0  0  0  0  0  0  0  0  0  0  0  0  0  0  0  0  0  0  0  0  0  0  0  0  0  0  0  0  0  0 |

Table2：the information of Nao Luo Tong capsule (NLTC) from ten different manufacturers

| number | SFDA approval number（ State Food and Drug Administration） | Manufacturer | lot number | China drug electronic supervision code |
| --- | --- | --- | --- | --- |
| 1 | Z44020002 | Guangzhou Baiyunshan Guanghua Pharmaceutical Co., Ltd. | T70074 | 81466630302529533891 |
| 2 | Z45021944 | Guangxi Huanbao Pharmaceutical Co., Ltd. | 20171002 | 6901247000614 |
| 3 | Z19993108 | Henan Furentang Pharmaceutical Co., Ltd. | 20171101 | 6938237700360 |
| 4 | Z44021943 | Dongguan Asia Pharmaceutical Co., Ltd. | 20160703 | 81347160004770435901 |
| 5 | Z44021297 | Zhaoqing Xinghu Pharmaceutical Co., Ltd. | 161202 | 6924604868399 |
| 6 | Z44023216 | Guangdong Yililu Custom Pharmaceutical Co., Ltd. | 160702 | 81590530028695552497 |
| 7 | Z22024825 | Tonghua Maoxiang Pharmaceutical Co., Ltd. | 170703 | 6938817515841 |
| 8 | Z19993103 | Jiaozuo Furuitang Pharmaceutical Co., Ltd. | 170807 | 6952608200778 |
| 9 | Z44022026 | Guangdong Bangmin Pharmaceutical Factory Co., Ltd. | 17116003 | 6922261201023 |
| 10 | Z20063131 | Diaoyutai Pharmaceutical Group Jilin Tian Compulsory Medicine Co., Ltd. | 160801086 | 6924653113785 |

Table3 heart tissue scoring criteria

| Standard | Score |
| --- | --- |
| The horizontal stripes are clear, the core is in the middle, and the interstitial is a small amount of blood vessels and connective tissue | 0 |
| Myocardial interstitial widening, there is light red dyed edema fluid | 1 |
| Small focal cardiomyocyte degeneration, necrosis, myocardial cell sarcoplasmic lysis or vacuolization, local inflammatory cell infiltration | 2 |
| The cytoplasm of the scattered cardiomyocytes is lightly stained, the striate is unclear, the cytoplasm is vacuolated, and even disintegrates. The cells are degenerated and necrotic under high magnification, and the local tissue cells and mononuclear macrophages increase | 3 |

Table4 Liver tissue scoring criteria

| Standard | Score |
| --- | --- |
| Hepatocytes in the central vein and its surroundings, hepatocytes are bulky, cytoplasm rich, eosinophilic | 0 |
| Hepatocyte volume increases, cytoplasm is loose and light, some have been necrotic, and there is small focal inflammatory cell infiltration | 1 |
| The volume increases, the cytoplasm is filled with tiny vacuoles, and some hepatocytes dissolve necrosis | 2 |
| The volume is increased, the cell boundary is unclear, and the cytoplasm is disintegrated into deep irritated red, irregularly shaped granules or small bodies | 3 |

Table5 Renal tissue scoring criteria

| Standard | Score |
| --- | --- |
| The glomerulus and surrounding renal tubules are clearly visible in the glomerular balloon lumen. The medullary collecting tube is a single-layered cubic epithelium with a cytoplasmic light-stained, nuclear round, centrally located. | 0 |
| The tubular epithelial cells increased in volume, the cytoplasm was red-stained, the lumen was irregular, and there were homogeneous red-dyed transparent casts, some of which were necrotic. The cytoplasm is loosely stained, the vacuole is changed, the cell boundary is unclear, the balloon cavity is dilated, and there is a foam-like liquid in the cyst cavity | 1 |
| The number of glomerular capillaries is reduced, the balloon cavity is dilated, there is foamy liquid in the cyst cavity, the epithelial hyperplasia of the balloon wall, degeneration and necrosis of renal tubular epithelial cells, vacuolization, unclear cell boundaries, red staining of cytoplasm, inflammatory cells Increased infiltration | 2 |
| The volume is increased, the cell boundary is unclear, and the cytoplasm is disintegrated into deep irritated red, irregularly shaped granules or small bodies | 3 |

Chemical Reference standards

| Product name | Cat.No | CAS No. | Molecular formula | Molecular weight | purity | Solubility |
| --- | --- | --- | --- | --- | --- | --- |
| Tolperisone(hydrochride) | HY.B1139 | 3644-61-9 | C16H24CINO | 281.82 | 99.78% | DMSO≥36mg/ml |
| Methyl Hesperidin | 111580 | 11013-97-1 | C29H36O15 | 624.59 | HPLC≥98% | Methanol, ethanol |
| Vitamin B6 | SV8110 | 58-56-0 | C8H12NO3CI | 205.64 | HPLC≥98% | water |

Figure 1.


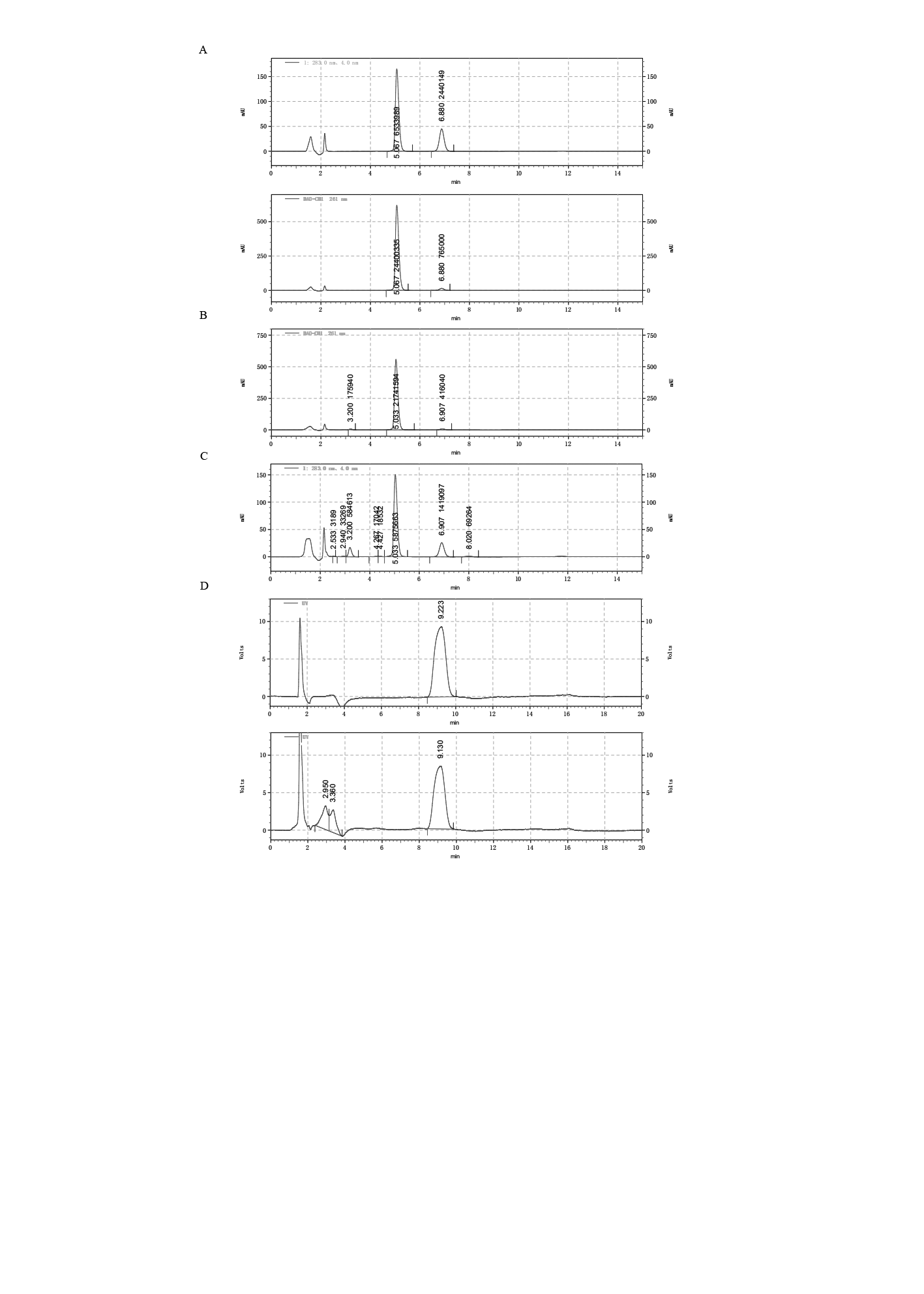


**Composition analysis of NLTC.** (A) mixed standards (methyl hesperidin and tolperisone hydrochloride) was detected at 283 nm (upper panel) and 261 nm (lower panel), respectively. (B) Methanol solution of NLTC was detected at wavelength of 261 nm. (C) Methanol solution of NLTC was detected at wavelength of 283 nm. (D) Vitamin B6 standard (upper panel) and Methanol solution of NLTC (lower panel) were detected at wavelength of 290 nm.
